# Streptococcus agalactiae-Related Splenic Abscess in Uncontrolled Diabetes Mellitus

**DOI:** 10.7759/cureus.9513

**Published:** 2020-08-01

**Authors:** Bipadabhanjan Mallick, Preetam Nath, Dibya L Praharaj, Sarat C Panigrahi, Anil Anand

**Affiliations:** 1 Gastroenterology, Kalinga Institute of Medical Sciences, Bhubaneswar, IND

**Keywords:** splenic abscess, streptococcus agalactiae, group b streptococcus

## Abstract

The spectrum of microorganisms causing splenic abscess is large, and commonly involved organisms include Enterobacteriaceae, gram-positive cocci and anaerobes. Group B Streptococcus (GBS) commonly causes infection in newborns and pregnant women, but there is increasing incidence of GBS causing invasive infection among nonpregnant adults, particularly among diabetics. Common presentations of GBS infection in adults include bacteremia, soft-tissue and skin infection, pneumonia, urinary tract infection, meningitis and endocarditis. We report a case of splenic abscess due to Streptococcus agalactiae infection without endocarditis in a diabetic patient.

## Introduction

Streptococcus agalactiae is a group B Streptococcus (GBS), and it commonly causes infection in newborns and pregnant women [1]. There is an increasing incidence of GBS causing invasive infection among nonpregnant adults, particularly among older persons and adults with diabetes [2]. Common presentations of GBS infection in adults include bacteremia, soft-tissue and skin infection, pneumonia, urinary tract infection, meningitis and endocarditis [2,3]. We report a case of splenic abscess due to Streptococcus agalactiae infection without endocarditis in a diabetic patient.

## Case presentation

**Figure 1:**
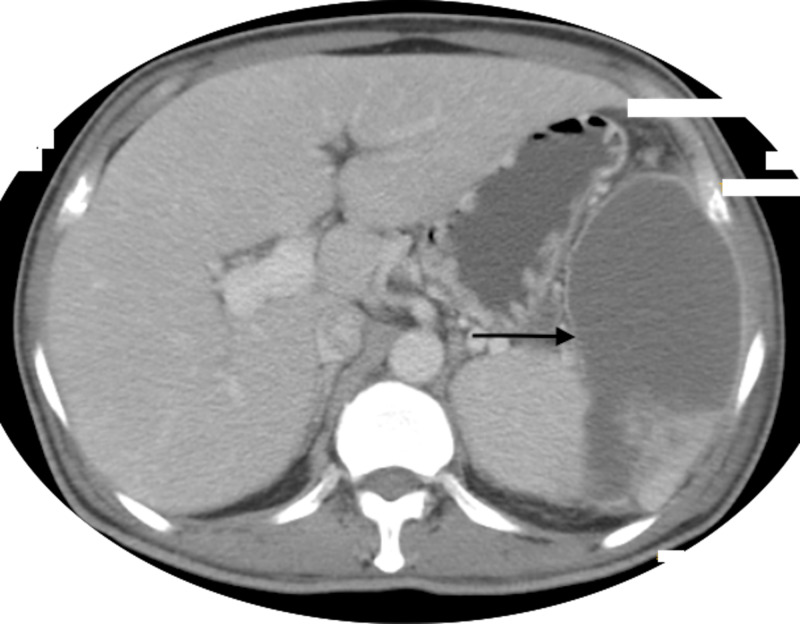
Contrast-enhanced CT of the abdomen showing splenic abscess (black arrow)

A 58-year-old man presented with a 20-day history of fever and pain in the left upper quadrant of abdomen. There was history of radiation of pain to the left shoulder. He had diabetes mellitus for 10 years with latest HbA1C value of 12.3%. Physical examination showed tender splenomegaly, and the patient’s laboratory workup revealed leukocytosis (23,000/mm^3^) with normal hemoglobin and platelets. He had normal liver and kidney function test. Serologies for human immunodeficiency virus, hepatitis B and hepatitis C were nonreactive. Ultrasonography of the abdomen showed splenomegaly with multiple hypoechoic collections, largest measuring 10 cm × 8 cm in diameter with shaggy borders, suggestive of an abscess. Contrast-enhanced CT of the abdomen confirmed the diagnosis of splenic abscess (Figure 1).

Minimally invasive pigtail catheter drainage was planned over splenectomy. A 12-Fr pigtail catheter was placed into the largest abscess cavity and the fluid culture grew Streptococcus agalactiae. Transthoracic and transesophageal echocardiography did not reveal any vegetation, and blood cultures were sterile. He recovered completely with a 14-day course of flucloxacillin (500 mg four times a day), and at three months of follow-up he was doing fine.

## Discussion

The spectrum of organisms causing splenic abscess is wide and has changed over time. Frequently involved microorganisms in splenic abscess are Enterobacteriaceae, gram-positive cocci and anaerobes [4]. Although GBS has been increasingly identified as a cause of invasive infections in nonpregnant adults, isolated splenic abscess due to GBS infection in the absence of infective endocarditis has not been reported in literature [1-5]. There are many underlying conditions that can lead to an increased risk for invasive GBS disease, including diabetes, cancer, chronic kidney disease, liver cirrhosis and immunosuppression [2,3]. Like our case, diabetes is the most common associated risk factor [2]. The choice of the treatment is similar to any other bacterial cause of splenic abscess, and it depends on the number, volume of abscesses and extrasplenic involvement [6,7]. Antibiotics therapy and interventional radiology have modified the therapeutic approach. Regarding antibiotic therapy for GBS infection, β-lactam antibiotics are considered to be the treatment of choice [8]. Splenectomy is usually considered in case of complications or when medical treatment is ineffective. 

## Conclusions

We reported the first case of splenic abscess caused by GBS infection in a nonpregnant adult patient without associated infective endocarditis. Culture-specific antibiotics combined with drainage with help of interventional radiology should be considered as the first line of therapy.
